# Cavitating leukoencephalopathy with multiple mitochondrial dysfunction syndrome and *NFU1* mutations

**DOI:** 10.3389/fgene.2014.00412

**Published:** 2014-11-20

**Authors:** Federica Invernizzi, Anna Ardissone, Eleonora Lamantea, Barbara Garavaglia, Massimo Zeviani, Laura Farina, Daniele Ghezzi, Isabella Moroni

**Affiliations:** ^1^Unit of Molecular Neurogenetics, Istituto Neurologico “Carlo Besta,” Fondazione Istituto di Ricovero e Cura a Carattere ScientificoMilan, Italy; ^2^Unit of Child Neurology, Istituto Neurologico “Carlo Besta,” Fondazione Istituto di Ricovero e Cura a Carattere ScientificoMilan, Italy; ^3^Unit of Neuroradiology, Istituto Neurologico “Carlo Besta,” Fondazione Istituto di Ricovero e Cura a Carattere ScientificoMilan, Italy

**Keywords:** leukoencephalopathy, multiple mitochondrial dysfunction syndrome, MMDS1, NFU1, mitochondrial disorders, brain MRI

## Abstract

Multiple Mitochondrial Dysfunction Syndrome (MMDS) comprises a group of severe autosomal recessive diseases with onset in early infancy and characterized by a systemic disorder of energy metabolism, resulting in weakness, respiratory failure, lack of neurological development, lactic acidosis, and early death. Biochemical findings include defects of complexes I, II, and III of the mitochondrial respiratory chain and severe deficiency of Pyruvate dehydrogenase complex (PDHc). Three genes have been associated with MMDS since now: *NFU1, BOLA3*, and *IBA57*. We describe an Italian male patient presenting with severe psychomotor regression after an infectious episode, lactic acidosis, hyperglycinemia, reduction of respiratory chain complex II associated with a marked deficiency of PDHc activity. He carried two heterozygous mutations in *NFU1*, one novel (p.Cys210Phe) and one previously reported (p.Gly189Arg) missense change affecting highly conserved residues. A severe leukoencephalopathy with cavitations in deep white matter was disclosed at brain MRI, suggesting a peculiar neuroradiological phenotype associated with defect in this gene.

## Introduction

Multiple Mitochondrial Dysfunction Syndrome (MMDS) is a severe autosomal recessive disease with onset in early infancy. It is characterized by a systemic disorder of energy metabolism resulting in marked impairment of neurologic development, with diffuse weakness, respiratory failure, lactic acidosis, and early death (Seyda et al., 2001; Roualt and Tong, 2008). MMDS has been associated with mutations in *NFU1* (causing MMDS1, MIM#605711), *BOLA3* (causing MMDS2, MIM#614299) and *IBA57* (causing MMDS3, MIM#615330) (Cameron et al., 2011; Navarro-Sastre et al., 2011; Ajit Bolar et al., 2013), all genes coding proteins with a role in iron-sulfur (Fe-S) cluster biosynthesis. Defects in this pathway, comprising also mutations in *ISCU* (MIM#255125) (Mochel et al., 2008), lead to abnormal function of many proteins containing Fe-S centers, including mitochondrial respiratory chain (MRC) complexes I, II, and III, and lipoic acid synthase (LIAS). The latter enzyme is needed for the maturation of four mitochondrial lipoate-dependent enzymes: pyruvate dehydrogenase complex (PDHc), α-ketoglutarate dehydrogenase (α-KGDH), branched-chain ketoacid dehydrogenase (BCKDH) and the H protein of the glycine cleavage system (MIM^*^238330) (Roualt and Tong, 2008; Stehling et al., 2014). Interestingly, mutations in *LIAS* cause Pyruvate dehydrogenase lipoic acid synthetase deficiency (MIM#614462), a syndrome characterized by several biochemical and clinical features overlapping with MMDS (Mayr et al., 2011).

NFU1 is a Fe-S cluster scaffold, initially considered as an alternative scaffold to ISCU, while recently characterized as a late targeting factor of Fe-S clusters, specifically required for the proper assembly of only a subset of (4Fe4S) proteins including LIAS and subunits of MRC complexes I and II (Cameron et al., 2011; Navarro-Sastre et al., 2011; Stehling et al., 2014). Since now three reports have described 14 patients belonging to 11 families with *NFU1* mutations (Cameron et al., 2011; Navarro-Sastre et al., 2011; Nizon et al., 2014): most cases presented with infantile encephalopathy associate with pulmonary hypertension, lactic acidosis, hyperglycinemia and severe regression leading to death before the age 15 months (Cameron et al., 2011; Navarro-Sastre et al., 2011). Only one child survived until the age of 2½ years (Nizon et al., 2014); leukoencephalopathy at brain MRI, has been illustrated only in this last patient, and reported without documentation in an additional case. Ten individuals from Spanish and French families were homozygous for p.Gly208Cys mutation, suggesting a founder effect in Europe. Other three mutations have been described in the remaining cases.

We describe the first Italian patient affected by MMDS1 associated with two heterozygous mutations in *NFU1*.

## Background

### Case report

**Figure 1:**
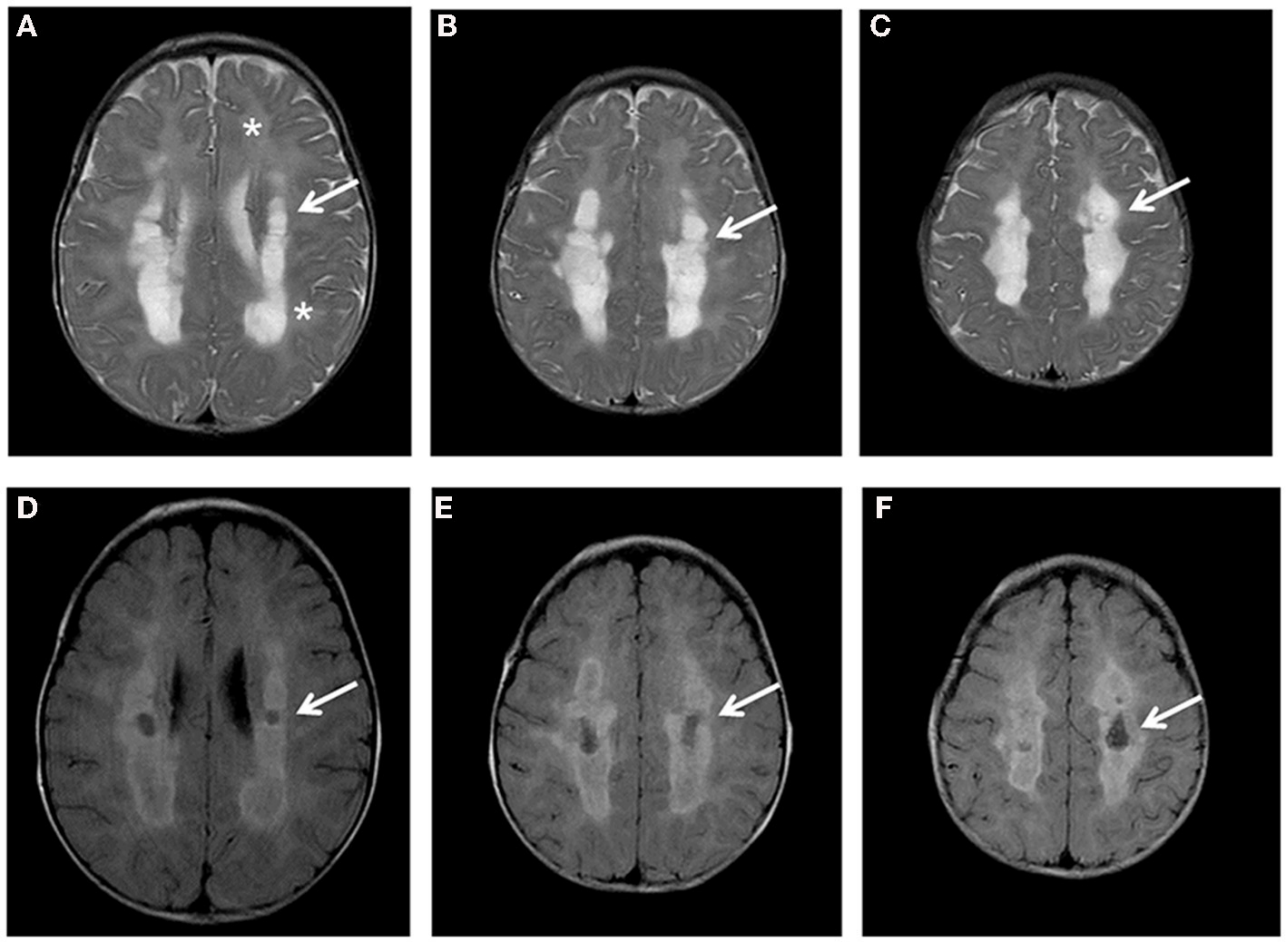
**Brain MRI**. Axial T2 **(A-C)** and FLAIR images **(D-F)** show diffuse hyper intensity of the white matter (**A**: asterisks) more prominent in the posterior periventricular and deep regions (**A-C**: arrows) with evidence of partial cystic degeneration and cavitations in FLAIR sections (**D-F**: arrows).

The proband was the first child born from unrelated Italian parents. Family history was negative; pregnancy and delivery were uneventful. Psychomotor development was referred normal until 7 months of age. During a mild febrile illness, he presented severe and rapid psychomotor regression, loosing sitting position and smiling, and developed spastic tetraparesis. Metabolic exams revealed high plasma lactate (5620 mmol/L; nv 580–2100 mmol/L) and pyruvate levels (294 mmol/L; nv 55–145), abnormal urinary excretion of lactic, succinic, fumaric, and glutaric acids; plasma amino acids analysis disclosed elevation of glycine (589 mmol/l, nv 11–360). Brain MRI showed the presence of a diffuse and symmetric involvement of hemispheric white matter with more marked abnormalities in the deep white matter that presented also cavitations (Figure 1). The cerebellum, brainstem and basal ganglia had normal appearance, while spectroscopy revealed a peak of lactic acid and decreased N-acetyl Aspartate/creatine ratio.

The neurological examination at 9 months revealed low body weight and height (10–25 centiles) with normal head circumference (50–75 centiles). He had good visual contact and response to sound, severe spasticity, absence of head and trunk control, dystonic postures, and frequent crying. Electroencephalogram showed a poor organization without epileptic abnormalities.

Electrocardiogram was normal and pulmonary hypertension was never detected. A mitochondrial disease was suspected and he underwent muscle and skin biopsies.

Despite the beginning of treatment with vitamin B2, B1, and Coenzyme Q the child further deteriorated during the following months: he suffered from several episodes of metabolic decompensation, developed epileptic seizures and required nasogastric feeding tube. He died at 18 months of age during a severe pneumonia; autopsy was not performed.

Muscle biopsy showed normal histological pictures. The analysis of MRC complexes activities revealed a clear reduction of complex II and SDH in muscle (43 and 48% of the control mean, respectively), and fibroblasts (30 and 55% respectively) and mild decrease of complex I in muscle (62%), and complex IV in fibroblasts (62%); the activate form of PDHc was also markedly reduced in both muscle and fibroblasts (28 and 1% respectively) (Table 1).

**Table 1:** **Biochemical activity of respiratory chain complexes and PDHc activities in muscle and fibroblasts**.

	**Muscle**	**Control range**	**Fibroblasts**	**Control range**
Complex I	**12.7**	13–28	28.1	10.7–26.0
Complex II	**9.2**	14–29	**4.0**	8.6–18.4
Complex III	104	65–125	89	70–120
Complex IV	169	130–250	**76**	95–150
Complex V	156	120–380	100	65–113
SDH	**7.1**	9.5–20.0	**5.7**	6.5–14.3
Citrate Synthase^*^	189	60–160	88	100–200
PDHc	**1.21**	2.70–6.00	**0.27**	2.40–3.60

*Values under the control range are reported in bold. All enzymatic activities are normalized for Citrate Synthase activity*.

* *nmol/min mg. SDH, succinate dehydrogenase; PDHc, pyruvate dehydrogenase complex*.

### Material and methods

Informed consent was obtained from patient's parents, in agreement with the Declaration of Helsinki and approved by the Ethical Committees of the Foundation IRCCS Istituto Neurologico “C. Besta,” Milan, Italy.

#### Biochemical studies

Skeletal muscle and skin biopsies were performed according to standard protocols. The measurement of MRC complexes activities was performed using standard spectrophotometric techniques (Bugiani et al., 2004) in both muscle homogenate and cultured skin fibroblasts, and normalized to citrate synthase (CS) activity, an index of mitochondrial content in the analyzed specimens. PDHc assay was performed with radioactive method (Uziel et al., 1988).

#### Molecular studies

DNA was extracted from peripheral blood of the patient and their parents using standard methods. The coding sequence and flanking intronic regions of *NFU1* were analyzed by PCR amplification using the primers reported in Supplementary Table 1. Amplicons were stained with ethidium bromide on 2% agarose gels, cycle-sequenced using BigDye chemistry 3.1, and run on an ABI 3130XL automatic sequencer (Applied Biosystems).

#### Western-blot analysis

Approximately 2 × 10^6^ fibroblasts were trypsinized, pelleted, sonicated, solubilized with 10 mM phosphate pH 7.2, 10 mM EDTA, 150 mM NaCl, 2% Triton X-100 and 0.25% SDS, in presence of a mixture of protease inhibitors, and centrifuged at 100,000 g for 30 min at 4°C. A 50 μg aliquot of protein per lane was then electrophoresed through an SDS-polyacrylamide gel. The gel was then electroblotted for 90 min onto a nitrocellulose filter. The latter was incubated with 5% non-fat milk in 20 mM Tris pH 8.0, 150 mM NaCl, 0.1% Tween-20 (MTT) for 60 min at room temperature followed by incubation overnight at 4°C with primary antibody. After four washing in MTT the filter was incubated for 60 min at room temperature with a goat anti-rabbit/mouse IgG conjugated to horseradish peroxidase (HRP) (1:5000 in MTT). After four additional washing, the peroxidase reaction was revealed by autoradiography using the chemiluminescence ECL kit (GE Healthcare). The following primary antibody, diluted in MTT, were used: α-NFU1 (Biorbyt, 1:50), α-NDUFA9 (subunit of complex I; Mitoscience, 1:2000), α-SDHA (subunit of complex II; Mitoscience, 1:10,000), α-SDHB (subunit of complex II; Mitoscience, 1:200), α-UQCRFS1 (subunit of complex III; Mitoscience, 1:1000), α-MTCO2 (subunit of complex IV; Mitoscience, 1:1500), α-COX4 (subunit of complex IV; Mitoscience, 1:2000), α-Tubulin beta (Sigma, 1:2000).

### Genetic and biochemical results

**Figure 2:**
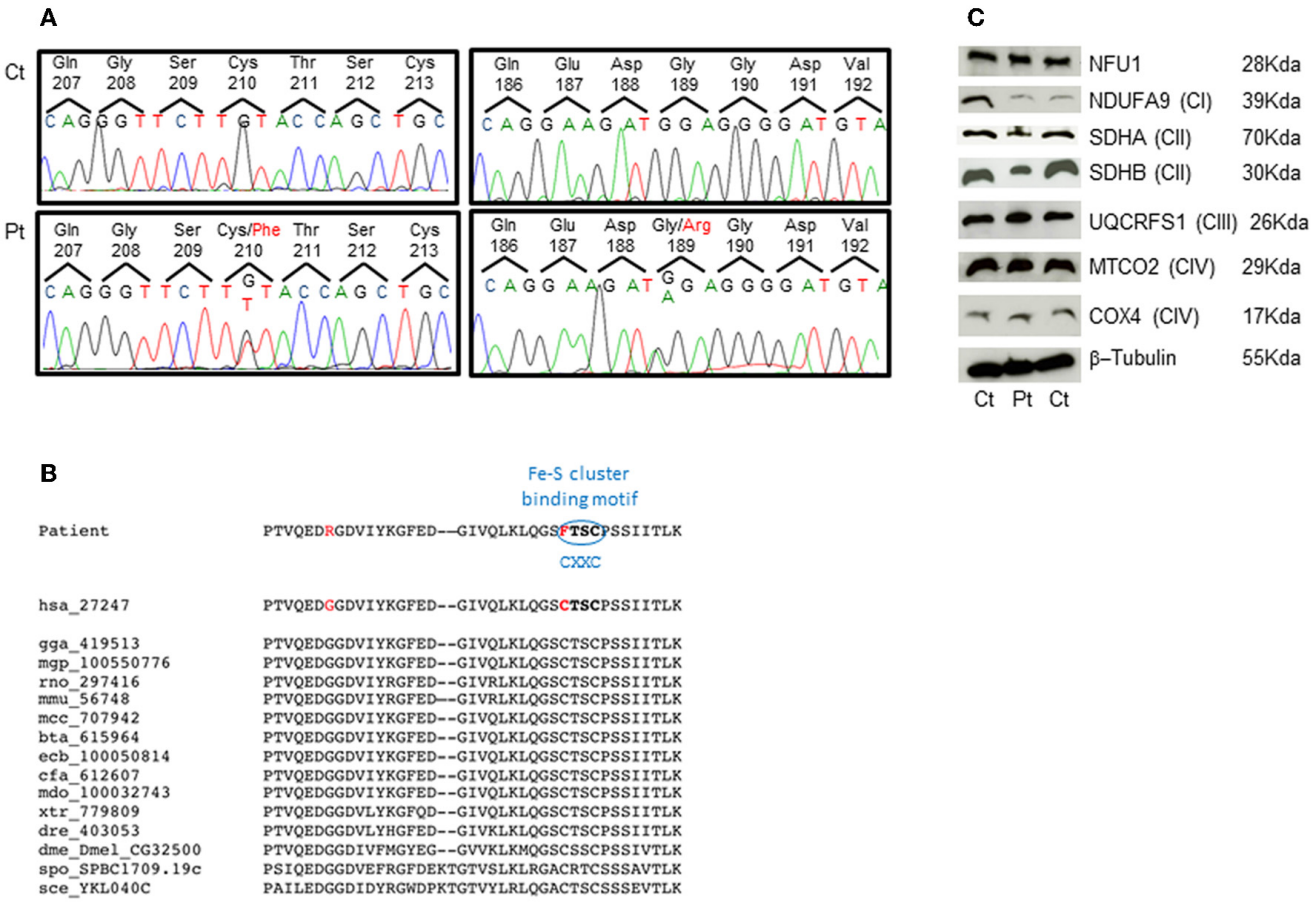
**Genetic studies and protein characterization in fibroblasts. (A)** Chromatograms of the *NFU1* genomic regions comprising the identified variant in the patient (Pt, lower panel) and in a control subject (Ct, upper panel). **(B)** Multiple alignment of NFU1 protein domain containing mutations. Both mutations affect highly conserved amino acids and the Cys210Phe strikes the first cysteine of the CXXC motif that is a characteristic binding domain for Fe-S cluster. **(C)** SDS-gel electrophoresis and Western blot analysis of fibroblasts from patient (Pt) and two controls (Ct). We used antibodies against NFU1; NDUFA9 (subunit of complex I); SDHA and SDHB (subunits of complex II); UQCRFS1 (subunit of complex III); MTCO2 and COX4 (subunits of complex IV); and β-Tubulin (as loading control).

The genetic analysis of *NFU1* showed the presence of two heterozygous mutations, c.565G>A and c.629G>T (NM_001002755.2), causing the p.Gly189Arg and p.Cys210Phe changes in the protein amino acid sequence (NP_001002755.1) (Figure 2A). Segregation analysis confirmed that the first mutation was inherited from the mother and the second from the father. The two identified nucleotide substitutions were not reported in public databases, including dbSNPs and NHLBI Exome Sequencing Project. The two affected amino acids are conserved in several species (Figure 2B) and the amino acid changes have high scores for pathogenicity according to different bioinformatic tools.

Western blot analysis performed on patient's fibroblasts showed no reduction of NFU1 protein; however, we found a partial reduction of the subunits of complex II, SDHA (70 kDa), and SDHB (30 kDa), whereas the amount of subunits belonging to other MRC complexes were similar to control levels (Figure 2C), using β-tubulin as loading control. These findings are in agreement with those recently reported by Ferrer-Cortes et al. (2013).

## Discussion

### Review similar cases

In 2001, Seyda et al. (2001) reported on three siblings, born from non-consanguineous parents of Mexican descent, affected with metabolic acidosis and high lactate levels, feeding difficulty, weakness and lethargy present since few days after birth; all three babies died within the first month of life. Biochemical studies demonstrated a combined defect of PDHc, MRC complex II, complex I + III, and complex II + III in fibroblasts from all patients. Laboratory investigations showed lactic acidosis and elevated levels of serum glycine.

Ten years later, next-generation sequencing (NGS) allowed Cameron et al. (2011) to detect a homozygous missense mutation (c.545G>A; p.Arg182Gln) in exon 6 of *NFU1* in these previously reported patients. Contemporary, Navarro-Sastre et al. (2011) reported ten individuals from nine unrelated Spanish families affected with fatal MMDS and carrying mutations in *NFU1* gene. In all patients the onset of symptoms started in the first months of life, and all died before the age of 16 months. They presented with failure to thrive, pulmonary hypertension, and neurological regression. For 3/13 published cases brain imaging data were available, and in only one case the presence of leukodystrophy was reported; the others were described with normal MRI pattern or unspecific and inconsistent features (e.g., cerebral atrophy, cerebellar lesions). However, white-matter necrosis with preservation of U fibers or areas of white-matter demyelination and vacuolization were found in cases in which autopsy was performed (Seyda et al., 2001; Navarro-Sastre et al., 2011). The biochemical phenotype included metabolic acidosis with variable lactic acidemia and hyperglycinemia, and high urinary excretion of 2-ketoglutaric, 2-ketoadipic, 2-hydroxyadipic, and glutaric acid. In the cases where tissues were available, activity of the glycine cleavage system in liver was low or undetectable. PDHc activity in fibroblasts was also decreased. The rates of ^14^C-substrate oxidation (pyruvate, leucine, and glutamate) in fibroblasts were low. Only in 3 patients MRC enzyme activities were assessed in frozen muscle, and a reduction of complex II + III activity (26–43% of control value) was detected. Genetic studies of *NFU1* in these patients showed the same c.622G>T/p.Gly208Cys mutation in exon 7 (9/10 homozygous, 1/10 heterozygous) and an additional intronic mutation c.545 + 5G>A affecting the donor splice-site of exon 6 (1/10 heterozygous). Recently, a further case was reported by Nizon et al. (2014), a French female with normal psychomotor development until 14 months of age, when she showed progressively losing of standing and sitting abilities with failure to thrive and pulmonary hypertension; then she developed a severe spastic tetraparesis associated with extrapyramidal signs. Lactate and pyruvate levels resulted within normal values, except during two episodes of metabolic decompensation. Glycine levels were high in plasma, urine and CSF, and urinary organic acid analysis showed mildly increased excretion of glutaric acid, 2-hydroxyglutaric acid and α-ketoglutaric acid. Fibroblasts analysis demonstrated a defect of PDHc activity (25%) and of complex II (28%). Brain MRI revealed the presence of leukoencephalopathy involving periventricular white matter and corpus callosum, that were partially cystic.

The clinical, biochemical and genetic data of the previous and present cases are reported in Table 2.

**Table 2:** **Clinical, biochemical and genetic findings in patients with *NFU1* mutations**.

**Family patient sex**	**Age at onset/outcome**	**Clinical features**	**MRI**	**Biochemical results**	**Enzyme activities**	***NFU1* nucleotide mutations**	**Amino acid change**	**References**
Family: 1	Birth / + <1 month	Feeding difficulties, weakness, lethargy	ND	Lactic acidosis, high serum glycine	Complex II, I + III, II + III and PDHc deficiency in fibroblasts	c.545G>A (3/3 homozygous)	p.Arg182Gln	Seyda et al., 2001; Cameron et al., 2011
Patients: 3								
2 M, 1 F								
Families: 9	2–9 months / + 2–15 months	7/10 pulmonary hypertension; 6/10 failure to thrive; 3/10 psychomotor retardation; 4/10 neurological regression	1/10 Leukodystrophy	Lactic acidosis, high serum and CSF glycine	Complex II + III and PDHc deficiency in fibroblasts	c.622G>T (9/10 homozygous, 1/10 heterozygous) c.545 + 5G>A (1/10 heterozygous)	p.Gly208Cys lack of mRNA expression	Navarro-Sastre et al., 2011
Patients: 10			1/10 Semioval center and cerebellum lesions					
7 F, 3 M			1/10 Cerebral atrophy					
Family: 1	5 months / alive 2½ yrs	Neurological regression, slow progression to spastic tetraparesis, dystonia, epilepsy	Leukoencephalopathy with periventricular and corpus callosum involvement and cystic degeneration	Lactate N in plasma, urine and CSF Glycine high in plasma, urine and CSF	Complex II, PDHc in fibroblasts	Compound heterozygous: c.622G>T c.565G>A	p.Gly189Arg	Nizon et al., 2014
Patient: 1							p.Gly208Cys	
1 F								
Family: 1	7 months / +18 months	Severe and rapid psychomotor regression, spastic tetraparesis, dystonic postures, epileptic seizures.	Leukoencephalopathy with periventricular involvement and cystic degeneration	Lactic acidosis, high serum glycine	Complex II, I, IV, PDHc deficiency in muscle and fibroblasts	Compound heterozygous: c.565G>A c.629G>T	p.Gly189Arg	Present paper
Patient: 1							p.Cys210Phe	
1 M								

*F, female; M, male, N, normal; PDHc, pyruvic dehydrogenase complex; +, age of death; ND, not done; CSF, cerebro spinal fluid; PDHc, pyruvate dehydrogenase complex*.

NFU1 is required for efficient assembly of lipoate synthase and respiratory complex II (SDH); NFU1 transiently bind the (4Fe–4S) cluster which may be transferred to the target apoprotein. The two mutations identified in our patient are both located in a highly conserved iron sulfur cluster assembly domain; moreover, the p.Cys210Phe change hits the first cysteine of a CxxC motif essential for the binding of Fe-S cluster. Although Western Blot analysis on fibroblasts failed to reveal any reduction of NFU1, the identified amino acid changes suggest a severe dysfunction of the protein, confirmed by the biochemical profile identified in our patients.

The presence of the p.Gly208Cys mutation in ten families from Spanish and French has suggested a founder effect in Europe (Navarro-Sastre et al., 2011; Nizon et al., 2014); the p.Gly189Arg mutation, found in our patient and in a previously described case (Nizon et al., 2014) can represent a second possible common European mutation.

The onset of symptoms and the clinical course in our case were similar to previously described patients, except for absence of pulmonary hypertension. The presence of abnormal urinary excretion of lactate and elevated levels of plasma glycine confirm that they represent the biochemical hallmarks of this syndrome.

Brain MRI pictures have not been fully described in the previous cases; the presence of white matter abnormalities has been reported in only one case from Navarro-Sastre series, but no MRI was available (Navarro-Sastre et al., 2011). Moreover, the evidence of a leukodystrophy associated with early development of cavitations in our patient similar to that described in one patient by Nizon et al. (2014), suggests that this may be the MRI specific pattern related to MMDS1. Interestingly, cavitating leukoencephalopathy represents an increasingly common MRI finding in mitochondrial disorders due to different genetic defects (Kashani et al., 2014; Melchionda et al., 2014).

## Concluding remarks

We have described the first Italian patient affected with MMDS1. This case contributes to delineate the phenotype associated with defects in *NFU1*, represented by severe early onset neurological impairment, multiple defects of respiratory chain and PDHc, high lactate and glycine levels. Moreover, the presence of leukodystrophy with cysts or cavitations is probably the commonest neuroradiological picture in MMDS1 patients.

### Conflict of interest statement

The authors declare that the research was conducted in the absence of any commercial or financial relationships that could be construed as a potential conflict of interest.
