# Supplementary material for: Cavitating leukoencephalopathy with multiple mitochondrial dysfunction syndrome and NFU1 mutations
Source: Front Genet. 2014 Nov 20;5:412. doi: 10.3389/fgene.2014.00412 (PMC4238403; doi:10.3389/fgene.2014.00412)
Supplement: Supplementary file 1 [file Table1.DOC]

Supplementary Table 1: Primers for *NFU1* exons amplification

| **Exon** | **Primer** | **Sequence** | **Annealing temperature** |
| --- | --- | --- | --- |
| 1 | NFU1-Ex 1A-F | gtaggccacc gcacttat | 54°C |
|  | NFU1-Ex 1A-R | gaggaagctccagagagt |  |
| 2 | NFU1-Ex 2-F | catgagaagt agaaaccaac | 52°C |
|  | NFU1-Ex 2-R | tcatagatctattggacaatatcc |  |
| 3 | NFU1-Ex 3-F | tgctttgtca gggcaataaa | 52°C |
|  | NFU1-Ex 3-R | gcagtgcacatagaaatgca |  |
| 4 | NFU1-Ex 4-F | gtgggattgc taaatcgtag | 53°C |
|  | NFU1-Ex 4-R | aagagagtaagaccctgtct |  |
| 5 | NFU1-Ex 5-F | aagatcacac cactgtactc | 52°C |
|  | NFU1-Ex 5-R | ctagattcctatgacttctg |  |
| 6 | NFU1-Ex 6-F | gtt tctgtcatca atttccc | 52°C |
|  | NFU1-Ex 6-R | tgtagagaagatacaggctt |  |
| 7 | NFU1-Ex 7-F | cttagccacagttattattc tg | 53°C |
|  | NFU1-Ex 7-R | tccagcccttgaagaaaaag |  |
| 8 | NFU1-Ex 8-F | aggataccgg gttatgattc | 53°C |
|  | NFU1-Ex 8-R | cttcaagttcctcagcatatta |  |

PCR conditions were 5 min at 94°C, followed by 30 cycles of 94°C for 30 s, annealing temperature (52, 53 or 54°C) for 30 s, 72°C for 45 s, and 72°C for 5 min.
